# Renal and Multiorgan Safety of ^177^Lu-PSMA-617 in Patients with Metastatic Castration-Resistant Prostate Cancer in the VISION Dosimetry Substudy

**DOI:** 10.2967/jnumed.123.265448

**Published:** 2024-01

**Authors:** Ken Herrmann, Kambiz Rahbar, Matthias Eiber, Richard Sparks, Nicholas Baca, Bernd J. Krause, Michael Lassmann, Walter Jentzen, Jun Tang, Daniela Chicco, Patrick Klein, Lars Blumenstein, Jean-René Basque, Jens Kurth

**Affiliations:** 1 Department of Nuclear Medicine, University of Duisburg-Essen and German Cancer Consortium, University Hospital Essen, Essen, Germany;; 2 Department of Nuclear Medicine, University Hospital Münster, Münster, Germany;; 3 Technical University of Munich, Munich, Germany;; 4 CDE Dosimetry Services, Knoxville, Tennessee;; 5 Department of Nuclear Medicine, Rostock University Medical Center, Rostock, Germany;; 6 Department of Nuclear Medicine, University Hospital Würzburg, Würzburg, Germany;; 7 Novartis Pharmaceuticals Corporation, East Hanover, New Jersey;; 8 Advanced Accelerator Applications, a Novartis Company, Turin, Italy;; 9 Novartis Institutes for BioMedical Research, East Hanover, New Jersey;; 10 Novartis Institutes for BioMedical Research, Basel, Switzerland; and; 11 Novartis Pharma AG, Basel, Switzerland

**Keywords:** ^177^Lu, prostate-specific membrane antigen, metastatic castration-resistant prostate cancer, radioligand therapy

## Abstract

In the VISION trial, [^177^Lu]Lu-PSMA-617 (^177^Lu-PSMA-617) plus protocol-permitted standard of care significantly improved overall survival and radiographic progression-free survival compared with standard of care alone in patients with prostate-specific membrane antigen–positive metastatic castration-resistant prostate cancer. This VISION dosimetry substudy quantified absorbed doses of ^177^Lu-PSMA-617 in the kidneys and other organs. **Methods:** Participants were a separate cohort of 30 nonrandomized patients receiving standard of care plus ^177^Lu-PSMA-617 at 7.4 GBq per cycle for up to 6 cycles. Blood samples, whole-body conjugate planar image scintigraphy, and abdominal SPECT/CT images were collected. SPECT/CT images were collected at 2, 24, 48, and 168 h after administration in cycle 1 and at a single time point 48 h after administration in cycles 2–6. Outcomes were absorbed dose per unit activity per cycle and cumulative absorbed dose over all cycles. Cumulative absorbed doses were predicted by extrapolation from cycle 1, and calculation of observed values was based on measurements of cycle 1 and cycles 2–6. Safety was also assessed. **Results:** Mean (±SD) absorbed doses per cycle in the kidneys were 0.43 ± 0.16 Gy/GBq in cycle 1 and 0.44 ± 0.21 Gy/GBq in cycles 2–6. The observed and predicted 6-cycle cumulative absorbed doses in the kidneys were 15 ± 6 and 19 ± 7 Gy, respectively. Observed and predicted cumulative absorbed doses were similar in other at-risk organs. Safety findings were consistent with those in the VISION study; no patients experienced renal treatment-emergent adverse events of a grade higher than 3. **Conclusion:** The renal cumulative absorbed ^177^Lu-PSMA-617 dose was below the established limit. ^177^Lu-PSMA-617 had a good overall safety profile, and low renal radiotoxicity was not a safety concern. Cumulative absorbed doses in at-risk organs over multiple cycles can be predicted by extrapolation from cycle 1 data in patients with metastatic castration-resistant prostate cancer receiving ^177^Lu-PSMA-617.

## Visual Abstract

**Figure:**
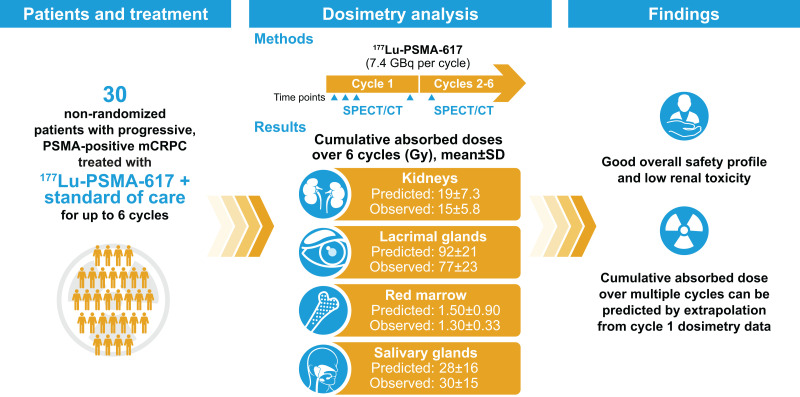

Radioligand therapy selectively targets cell-surface proteins expressed on cancer cells and spares most normal tissues (1). Prostate-specific membrane antigen (PSMA) is highly expressed in prostate cancer cells, with limited expression in nonprostate cancer cells (2–4). Radioligand therapies targeting PSMA are promising new treatments for patients with metastatic castration-resistant prostate cancer (mCRPC) (5–7).

[^177^Lu]Lu-PSMA-617 (^177^Lu-PSMA-617) is a high-affinity PSMA-targeted small-molecule radioligand therapy that delivers β-particle radiation specifically to prostate cancer lesions (8–10). The randomized, open-label, pivotal, phase 3 VISION study showed that ^177^Lu-PSMA-617 prolonged radiographic progression-free survival (hazard ratio, 0.40; 99.2% CI, 0.29–0.57; *P* < 0.001) and overall survival (hazard ratio, 0.62; 95% CI, 0.52–0.74; *P* < 0.001) when added to protocol-permitted standard of care in patients with advanced PSMA-positive mCRPC (11). The incidence of adverse events of grade 3 or above was higher with ^177^Lu-PSMA-617 than without, but health-related quality of life and pain were not adversely affected (11).

The kidneys have long been recognized as dose-limiting organs for therapeutic radiopharmaceuticals, with a historical cumulative absorbed dose limit of 23 Gy (12,13). This limit is based on external beam radiation therapy (EBRT) and has not been revised on the basis of experience with systemic radiopharmaceuticals. In patients receiving ^177^Lu-PSMA-617, the kidneys are exposed to radiation because urinary excretion is the principal route of elimination and because PSMA is expressed in proximal tubular cells (14). Other organs at risk of radiotoxicity with ^177^Lu-PSMA-617 include the lacrimal glands, salivary glands, and red marrow (15–17). The lacrimal and salivary glands are at risk because of physiologic uptake due to a combination of PSMA-specific and nonspecific mechanisms (18,19). Red marrow is at risk because reserve is often depleted by previous cytotoxic therapies (20) and radiation-induced myelosuppression can occur. Characterizing the biodistribution of ^177^Lu-PSMA-617 in a dosimetry study is therefore crucial for informed assessment of the risk of radiation-induced adverse events (13,21). Similar to EBRT, dosimetry studies of radiopharmaceutical therapies should be performed to improve the understanding of the effects of radiation exposure. Some countries have therefore made dosimetry studies a legal requirement (22,23).

The VISION study included a dosimetry substudy that aimed to enhance the assessment of the safety profile of ^177^Lu-PSMA-617 and to contextualize exposure levels against historical EBRT limits in patients with mCRPC. We used a simplified dosimetry approach based on conventional multiple-time-point imaging in cycle 1 and single-time-point imaging in cycles 2–6 (24). We report absorbed radiation doses in kidneys and other organs, as well as safety and tolerability findings and pharmacokinetic data. We also investigated whether cumulative absorbed radiation doses over multiple cycles of ^177^Lu-PSMA-617 treatment can be predicted by extrapolation from cycle 1 dosimetry data.

## MATERIALS AND METHODS

### Patients

A separate nonrandomized cohort of 30 eligible patients was enrolled into the dosimetry substudy at 4 sites in Germany. The patient selection criteria were the same as for the pivotal VISION study (11). Full details are provided in the supplemental materials (available at http://jnm.snmjournals.org).

### Treatment

In addition to protocol-permitted standard of care, all patients in the substudy received ^177^Lu-PSMA-617 (7.4 GBq, 200 mCi) per cycle every 6 wk for up to 6 cycles. The treatment regimen and patient management were identical to those in the pivotal VISION study (11).

### Objectives

The primary objectives were to conduct whole-body and organ dosimetry of ^177^Lu-PSMA-617 and to characterize its biodistribution. We investigated whether cumulative absorbed doses extrapolated from cycle 1 dosimetry data could predict observed cumulative absorbed doses for multiple cycles of ^177^Lu-PSMA-617 treatment. Analyses focused on organs at risk of radiotoxicity from ^177^Lu-PSMA-617 (kidneys, lacrimal glands, salivary glands, and red marrow).

The secondary objectives were to evaluate the safety and tolerability of ^177^Lu-PSMA-617, to evaluate cardiac function during treatment, to define the pharmacokinetic profile of ^177^Lu-PSMA-617, and to characterize the radiometabolites of ^177^Lu-PSMA-617 in urine.

Results were analyzed descriptively and separately from those of the pivotal VISION study.

### Image Acquisition and Dosimetry

**FIGURE 1.**
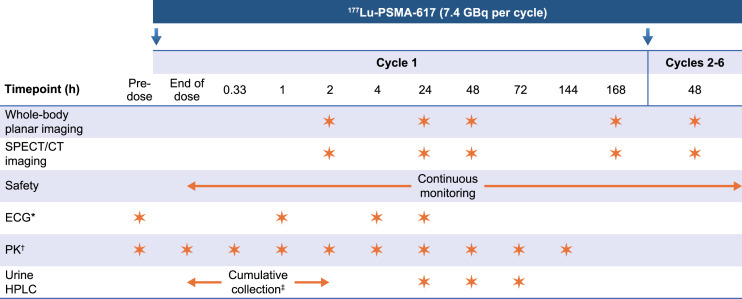
Study design and assessments. Asterisk shows that blood pressure was measured before each electrocardiogram (ECG). Dagger symbol is blood pharmacokinetic (PK) samples that were collected after ECGs when time points overlapped. Double dagger shows whole urine collection that was required between end of dose and 2 h after dose before first image. HPLC = high-performance liquid chromatography.

Whole-body conjugate planar image scintigraphy and abdominal SPECT/CT images were collected at 2, 24, 48, and 168 h after administration in cycle 1 and at a single time point 48 h after administration in cycles 2–6 (Fig. 1). This was selected as the most convenient single time point lying in the center of the complete curve collected in cycle 1. Technical details of the used imaging systems and applied acquisition and reconstruction protocols are shown in Supplemental Table 1 (25). Whole-body and specific organ-absorbed doses (26–28) for cycles 2–6 were derived using single-time-point data and individual time–activity curves generated for cycle 1. Blood samples were collected in cycle 1 only, at time points immediately before administration, immediately after administration (assigned as 1 min), and at approximately 20 min and 1, 2, 4, 24, 48, 72, and 144 h after administration (Fig. 1). Red marrow–absorbed doses were based on an assay of blood samples and the remainder-of-body activity from cycle 1 using the standard blood-based methodology (29). Absorbed doses were estimated using OLINDA/EXM software version 2.2.2 (Hermes Medical Solutions). Lacrimal gland dosimetry used the MIRD/Radiation Dose Assessment Resource method (17,30–37).

Dosimetry outcomes were absorbed dose per unit activity (Gy/GBq) during cycle 1 and cycles 2–6 and predicted and observed cumulative absorbed dose (Gy) over all 6 cycles (44.4 GBq). Cumulative absorbed doses were predicted by extrapolation from multiple-time-point cycle 1 data in all patients, and calculation of observed values was based on multiple-time-point cycle 1 data and measurements of additional single-time-point cycles 2–6. Predicted and observed cumulative absorbed doses were compared statistically using Hotelling T-squared tests with Bonferroni adjustment for multiple comparisons. Full details are provided in the supplemental materials.

### Safety and Tolerability

Treatment-emergent adverse events (TEAEs) were monitored throughout the substudy and were defined as those occurring from the first administration of treatment up to and including 30 d after the last administration or before receipt of subsequent anticancer treatment, whichever occurred first. TEAEs in the renal toxicity safety topic of interest were increased blood creatinine level, acute kidney injury, increased blood urea level, proteinuria, kidney failure, and decreased urine output. TEAEs in the nausea and vomiting safety topic of interest were nausea, vomiting, and retching. TEAEs in the bone marrow suppression safety topic of interest were anemia, thrombocytopenia, lymphopenia, leukopenia, neutropenia, pancytopenia, febrile neutropenia, bicytopenia, bone marrow failure, and normocytic anemia. TEAEs in the dry mouth safety topic of interest were dry mouth, aptyalism, dry lips, and dry throat. TEAEs in the hepatotoxicity safety topic of interest were increased aspartate aminotransferase, increased blood alkaline phosphatase, hypoalbuminemia, increased alanine aminotransferase, hyperbilirubinemia, ascites, increased γ-glutamyltransferase, acute hepatic failure, cholestasis, hepatic encephalopathy, hepatic failure, hepatic lesion, hepatitis, hepatocellular injury, increased international normalized ratio, jaundice, and increased transaminases. TEAEs were assessed using the Common Terminology Criteria for Adverse Events (CTCAE) version 5.0.

### Study Conduct

The VISION study was registered on ClinicalTrials.gov (NCT03511664) and was conducted in accordance with the principles of the Declaration of Helsinki, the International Conference on Harmonization Good Clinical Practice, and applicable local regulations. Independent ethics review boards approved the trial protocol at each trial site. All patients in the pivotal study and the present dosimetry substudy signed written informed consent forms before enrollment.

## RESULTS

### Patients

All 30 enrolled patients received at least 1 cycle of ^177^Lu-PSMA-617 plus standard of care. In cycle 1, multiple-time-point dosimetry was performed in 29 patients—1 patient received ^177^Lu-PSMA-617 but was unable to tolerate the imaging procedures required for dosimetry because of intense bone pain. One patient underwent imaging at 24 h in cycle 2 for clinical reasons and then at 48 h in all subsequent cycles. In addition, single-time-point dosimetry was performed in 21 patients who completed cycles 2 and 3, in 19 patients who completed cycle 4, in 13 patients who completed cycle 5, and in 10 patients who completed all 6 cycles.

A total of 18 patients (60.0%) discontinued all study treatments. Reasons for discontinuation of ^177^Lu-PSMA-617 were disease progression (*n* = 7), investigator decision (*n* = 4), adverse events (*n* = 3), withdrawal of consent to treatment (*n* = 2), death (*n* = 2), lack of clinical benefit (*n* = 1), and other (*n* = 1).

### Dosimetry

#### Absorbed Doses per Cycle

**FIGURE 2.**
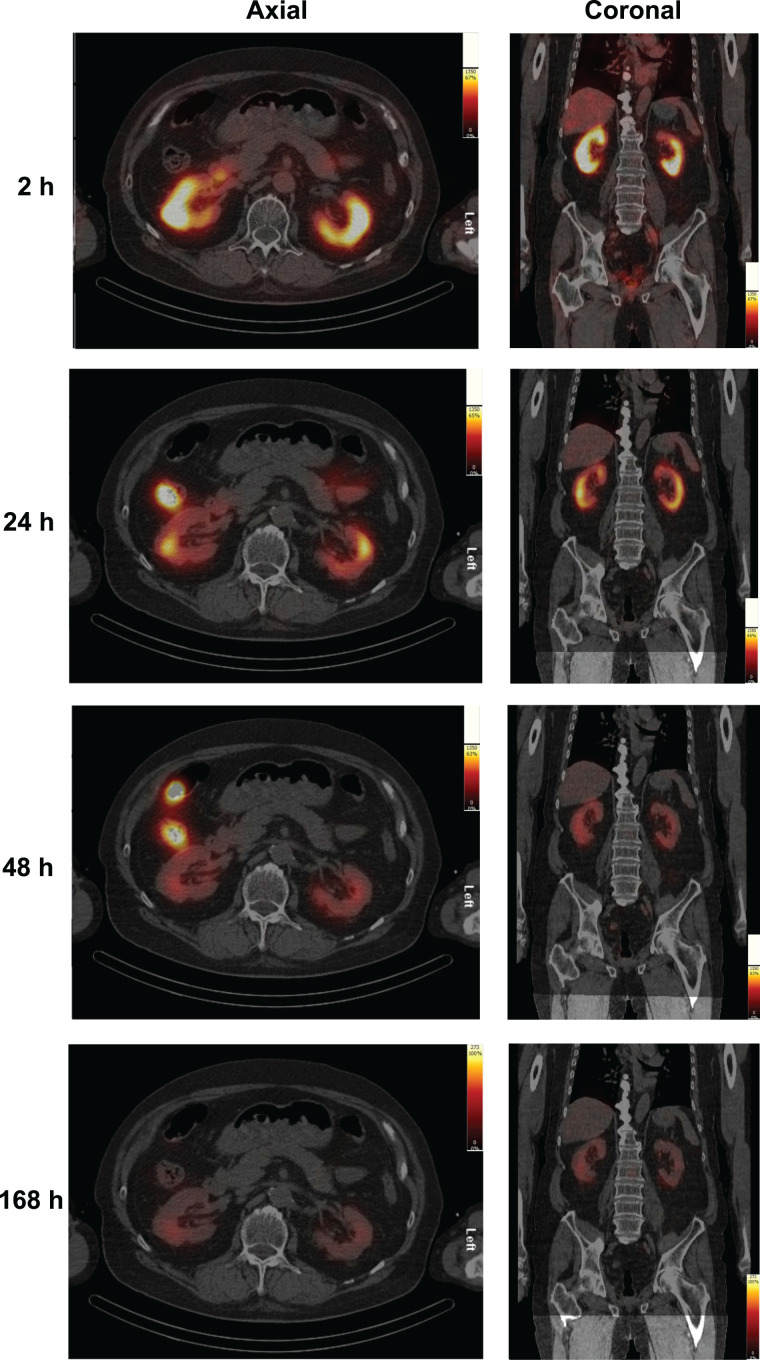
Representative SPECT/CT images show kidneys of a patient at various times during cycle 1 of ^177^Lu-PSMA-617 treatment. SPECT images (black/red/yellow scale) show uptake of ^177^Lu-PSMA-617 in kidneys (axial and coronal orientations) at 2, 24, 48, and 168 h during cycle 1. Underlaid CT images (gray) are scaled equally. Both image types are maximum-intensity projections.

In cycle 1, absorbed doses per unit activity among at-risk organs were highest in the lacrimal and salivary glands, with mean values ± SD of 2.10 ± 0.47 and 0.63 ± 0.36 Gy/GBq, respectively, followed by the kidneys at 0.43 ± 0.16 Gy/GBq and red marrow at 0.035 ± 0.020 Gy/GBq (Table 1). Figure 2 shows representative SPECT/CT images of the kidneys of a patient taken at 4 time points during cycle 1. Supplemental Figure 1 shows representative contouring of the kidneys on SPECT/CT images taken during cycle 1.

**TABLE 1.** Absorbed Doses per Unit Activity per Cycle

	Cycle 1*		Cycles 2–6*	
Organ or tissue	Mean	SD	Mean	SD
**Lacrimal glands**	**2.1 (1.2–3.2)**	**0.47**	**1.8 (0.70–3.9)**	**0.61**
**Salivary glands**	**0.63 (0.22–1.5)**	**0.36**	**0.63 (0.23–1.4)**	**0.30**
Left colon	0.58 (0.33–1.0)	0.14	0.58 (0.32–0.73)	0.11
Rectum	0.56 (0.32–1.1)	0.14	0.55 (0.31–0.70)	0.10
**Kidneys**	**0.43 (0.22–0.83)**	**0.16**	**0.44 (0.17–1.0)**	**0.21**
Right colon	0.32 (0.18–0.60)	0.08	0.31 (0.18–0.40)	0.06
Urinary bladder wall	0.32 (0.29–0.43)	0.03	0.33 (0.29–0.43)	0.03
Thyroid	0.26 (0.09–1.69)	0.37	0.21 (0.06–1.6)	0.25
Heart wall	0.17 (0.03–0.52)	0.12	0.15 (0.05–0.37)	0.08
Lungs	0.11 (0.03–0.57)	0.11	0.06 (0.02–0.17)	0.03
Liver	0.090 (0.043–0.220)	0.044	0.11 (0.037–0.26)	0.054
Small intestine	0.071 (0.043–0.220)	0.031	0.065 (0.043–0.083)	0.010
Spleen	0.067 (0.031–0.140)	0.027	0.095 (0.028–0.32)	0.056
Osteogenic cells	0.036 (0.02–0.170)	0.028	0.030 (0.016–0.062)	0.009
**Red marrow**	**0.035 (0.020–0.13)**	**0.020**	**0.031 (0.021–0.051)**	**0.007**
Adrenal glands	0.033 (0.016–0.15)	0.025	0.028 (0.014–0.060)	0.009
Gallbladder wall	0.028 (0.013–0.15)	0.026	0.023 (0.012–0.055)	0.008
Pancreas	0.027 (0.012–0.15)	0.026	0.021 (0.008–0.051)	0.008
Prostate	0.027 (0.013–0.15)	0.026	0.021 (0.007–0.050)	0.008
Esophagus	0.025 (0.010–0.15)	0.026	0.019 (0.006–0.050)	0.008
Stomach wall	0.025 (0.011–0.15)	0.026	0.019 (0.006–0.050)	0.008
Thymus	0.025 (0.010–0.15)	0.026	0.018 (0.004–0.049)	0.008
Testes	0.023 (0.010–0.14)	0.025	0.017 (0.003–0.046)	0.008
Eyes	0.022 (0.009–0.14)	0.024	0.016 (0.003–0.045)	0.008
Brain	0.007 (0.002–0.025)	0.005	0.006 (0.003–0.028)	0.003
Whole body	0.037 (0.019–0.170)	0.027	0.031 (0.018–0.065)	0.009

* Dosimetry data were available for 29 patients at cycle 1, 21 patients at cycles 2 and 3, 19 patients at cycle 4, 13 patients at cycle 5, and 10 patients at cycle 6.

Bold font indicates organs considered to be at particular risk of radiotoxicity. Data are Gy/GBq.

In cycles 2–6, absorbed doses per unit activity per cycle among at-risk organs were similar to those in cycle 1, with the highest mean values in the lacrimal and salivary glands at 1.80 ± 0.61 and 0.63 ± 0.30 Gy/GBq, respectively, followed by the kidneys at 0.44 ± 0.21 Gy/GBq and red marrow at 0.031 ± 0.007 Gy/GBq (Table 1).

Table 1 shows absorbed doses in all organs and tissues assessed. Average percent injected activity is shown in Supplemental Figure 2 and Supplemental Table 2. The normalized number of disintegrations in specific organs is shown in Supplemental Table 3.

#### Cumulative Absorbed Doses

Predicted 6-cycle cumulative absorbed doses based on extrapolation of cycle 1 data in 29 patients were 92.0 ± 21.0 Gy in the lacrimal glands, 28.0 ± 16.0 Gy in the salivary glands, 19.0 ± 7.3 Gy in the kidneys, and 1.5 ± 0.9 Gy in the red marrow (Table 2). Observed 6-cycle cumulative absorbed doses in the 10 patients who completed all 6 cycles were 77 ± 23 Gy in the lacrimal glands, 30 ± 15 Gy in the salivary glands, 15 ± 6 Gy in the kidneys, and 1.30 ± 0.33 Gy in the red marrow (Table 2). Table 2 shows cumulative doses in all organs and tissues assessed. Supplemental Table 4 shows predicted and observed cumulative absorbed doses in the 10 patients who completed all 6 cycles of treatment.

**TABLE 2.** Cumulative Absorbed Doses Over 6 Cycles

	Predicted from cycle 1 data (*n* = 29)		Observed (*n* = 10)		
Organ or tissue	Mean	SD	Mean	SD	Relative difference
**Lacrimal glands**	**92 (54–140)**	**21**	**77 (53–115)**	**23**	**+19.5%**
**Salivary glands**	**28 (10–68)**	**16**	**30 (11–58)**	**15**	**−6.7%**
Left colon	26 (15–45)	6.0	24 (14–29)	4.8	+8.3%
Rectum	25 (14–47)	6.2	23 (13–28)	4.6	+8.7%
**Kidneys**	**19 (10–37)**	**7.3**	**15 (9.1–29)**	**5.8**	**+26.7%**
Right colon	14 (8.1–27)	3.4	13 (8.0–16)	2.5	+7.7%
Urinary bladder wall	14 (13–19)	1.1	14 (13–15)	0.58	0.0%
Thyroid	11 (3.8–75)	16	9.8 (3.3–48)	14	+12.2%
Heart wall	7.8 (1.4–23)	5.2	5.7 (2.8–11)	3.0	+36.8%
Lungs	4.7 (1.3–25)	4.9	2.5 (1.2–5.4)	1.3	+88.0%
Liver	4.0 (1.9–9.6)	2.0	4.0 (2.1–9.3)	2.1	0.0%
Small intestine	3.1 (1.9–9.9)	1.4	2.7 (2.2–3.1)	0.36	+14.8%
Spleen	3.0 (1.4–6.0)	1.2	3.4 (1.4–8.0)	2.3	−11.8%
Osteogenic cells	1.6 (0.88–7.6)	1.3	1.3 (0.85–2.2)	0.44	+23.1%
Adrenal glands	1.5 (0.70–6.8)	1.1	1.1 (0.62–2.0)	0.41	+36.4%
**Red marrow** *	**1.5 (0.87–5.9)**	**0.9**	**1.3 (0.93–1.8)**	**0.33**	**+15.4%**
Gallbladder wall	1.2 (0.56–6.7)	1.1	0.96 (0.52–1.8)	0.40	+25.0%
Pancreas	1.2 (0.55–6.7)	1.1	0.90 (0.50–1.8)	0.38	+33.3%
Prostate	1.2 (0.59–6.7)	1.1	0.91 (0.54–1.8)	0.36	+31.9%
Esophagus	1.1 (0.46–6.5)	1.1	0.81 (0.42–1.7)	0.39	+35.8%
Stomach wall	1.1 (0.48–6.6)	1.1	0.83 (0.44–1.7)	0.38	+32.5%
Thymus	1.1 (0.45–6.5)	1.1	0.78 (0.41–1.7)	0.39	+41.0%
Testes	1.0 (0.43–6.3)	1.1	0.74 (0.39–1.6)	0.36	+35.1%
Eyes	1.0 (0.40–6.1)	1.1	0.72 (0.36–1.6)	0.36	+38.9%
Brain	0.3 (0.08–1.1)	0.2	0.27 (0.17–0.41)	0.08	+11.1%
Whole body	1.6 (0.86–7.3)	1.2	1.3 (0.79–2.2)	0.42	+23.1%

* Cycles 2–6 observed data were based on blood samples and remainder-of-body activity from cycle 1 scaled according to respective imaging data. For cycle 6, total injected activity was 44.4 GBq.

Data are Gy. Bold font indicates organs considered to be at particular risk of radiotoxicity.

**FIGURE 3.**
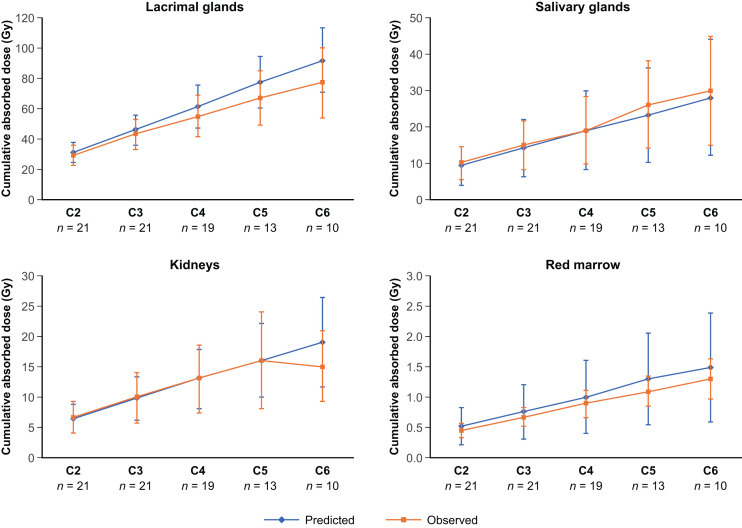
Predicted versus observed cumulative absorbed doses per cycle in at-risk organs. Injected activity: cycle 2, 14.8 GBq; cycle 3, 22.2 GBq; cycle 4, 29.6 GBq; cycle 5, 37.0 GBq; cycle 6, 44.4 GBq.

Predicted versus observed cumulative absorbed doses per cycle in at-risk organs were similar to each other at both the group level and the individual patient level (Fig. 3; Supplemental Fig. 3). For each cycle in cycles 2–6, the predicted values were similar to observed values, independent of the number of patients with data for subsequent cycles. Statistical comparisons of predicted versus observed cumulative doses across all 4 at-risk organs revealed no significant differences (*P* = 0.19–0.54 in cycles 2–6). Predicted values were generally higher in later treatment cycles compared with the observed values (Fig. 3; Table 2).

### Safety and Tolerability

The median duration of ^177^Lu-PSMA-617 exposure was 5.52 mo (range, 1.4–9.0 mo), with patients receiving a median of 4 cycles and a mean total administered activity of 28.7 ± 10.5 GBq. Across all treatment cycles, similar proportions of patients in this substudy experienced TEAEs as in the pivotal VISION study, including severe, serious, and drug-related adverse events (Supplemental Table 5). TEAEs grouped as safety topics of interest are shown in Table 3. Renal toxicity was reported in 5 of 30 patients (16.7%), of whom 3 of 30 (10%) had increased creatinine levels. The mean cumulative absorbed dose in the kidneys over 6 cycles for patients with reported renal toxicity was 24.42 ± 7.41 Gy versus 17.55 ± 7.80 Gy in those without (based on observed dose when available and on predicted dose when observed dose was not available). No patients experienced renal TEAEs of grade 3 or higher. TEAEs were most frequent in the bone marrow suppression grouping, occurring in 11 of 30 patients (36.7%), of whom 2 of 30 patients (6.7%) had grade 4 events (lymphopenia and thrombocytopenia), 1 of 30 patients (3.3%) had grade 3 events (leukopenia and thrombocytopenia), and 2 of 30 patients (6.7%) had grade 3 events (anemia). TEAEs were also frequent in the nausea and vomiting grouping, occurring in 11 of 30 patients (36.7%) each, followed by fatigue in 6 of 30 patients (20%) and dry mouth in 5 of 30 patients (16.7%) (Table 3).

**TABLE 3.** TEAEs Grouped as Safety Topics of Interest in the Substudy

	^177^Lu-PSMA-617 plus SoC (*n* = 30)	
Safety topic	All grades	Grade ≥ 3 (%)
Bone marrow suppression	11 (36.7)	6 (20.0)
Nausea and vomiting	11 (36.7)	0 (0.0)
Fatigue	6 (20.0)	2 (6.7)
Dry mouth	5 (16.7)	0 (0.0)
Renal toxicity	5 (16.7)	0 (0.0)
Hepatotoxicity	2 (6.7)	0 (0.0)
Intracranial hemorrhage	0 (0.0)	0 (0.0)
QT prolongation	0 (0.0)	0 (0.0)
Reproductive toxicity	0 (0.0)	0 (0.0)
Second primary malignancies	0 (0.0)	0 (0.0)

Toxicity was assessed using CTCAE version 5.0. Safety topics occurred on or after start of treatment with ^177^Lu-PSMA-617 plus standard of care (SoC) up to 30 d after last administration or before initiation of subsequent anticancer treatment. Patient with multiple grades for safety topic is counted only under maximum grade. Data in parentheses are percentages.

#### Toxicity Adverse Events in Cycle 1

During cycle 1, 6 of 30 patients (20%) had 1 or more hematologic adverse events of CTCAE grade 2 or higher, based on worsening from baseline (Supplemental Table 6). The most common of these was anemia in 5 of 30 patients (16.7%). No patients had thrombocytopenia of CTCAE grade 2 or higher.

During cycle 1, 2 patients (6.7%) had salivary gland adverse events, limited to CTCAE grade 1. No patients had lacrimal gland adverse events or renal toxicity during cycle 1 (Supplemental Table 6).

#### Cardiac Safety

Low uptake of ^177^Lu-PSMA-617 in the heart wall was observed, with an absorbed dose per unit activity of 0.17 ± 0.12 Gy/GBq in cycle 1 (Table 1).

Least-square mean changes in the corrected Fridericia QT interval from baseline were minimal, ranging from +2.1 to −5.2 ms during the first 24 h of treatment (Supplemental Fig. 4A). Least-square mean changes in the heart rate from baseline were also minimal, ranging from −0.8 to +3.8 bpm during the first 24 h of treatment (Supplemental Fig. 4B).

### Pharmacokinetics

#### Plasma Profile

After infusion, the peak ^177^Lu-PSMA-617 plasma concentration was generally reached approximately 20 min after administration (median maximum time, 0.38 h) and was followed by a biexponential decline (Supplemental Fig. 5).

The geometric mean half-life of ^177^Lu-PSMA-617 from the circulation was approximately 41.6 h (geometric mean coefficient of variation, 68.8%) (Supplemental Table 7).

#### Urinary Metabolites

Radioactivity in urine at 72 h was decreased by approximately 93% compared with the 0- to 2-h time point. The proportion of intact ^177^Lu-PSMA-617 in the urine decreased from approximately 95% of total urine radioactivity at 0–2 h to 92% at 24 h, 83% at 48 h, and 68% at 72 h, with radiometabolites accounting for the remaining total radioactivity. The most common radiometabolites in urine were M1, M3, and M4, with a total of 9 peaks (Supplemental Fig. 6).

## DISCUSSION

The cumulative dose limit of 23 Gy for the kidneys has been historically derived for EBRT and subsequently applied to radiopharmaceutical use (12,13). In this dosimetry substudy of the pivotal phase 3 VISION study of ^177^Lu-PSMA-617 in patients with mCRPC, absorbed doses in all cycles were within accepted limits in the kidneys and other organs at the highest risk of radiotoxicity. Absorbed doses per cycle were highest in the lacrimal and salivary glands, followed by the kidneys and the red marrow. The observed renal cumulative absorbed dose was 15 Gy at cycle 6, which was below the historical limit of 23 Gy (12,13). TEAEs affecting the kidneys and other organs at risk of radiotoxicity were infrequent and generally of low to moderate severity.

For each of cycles 2–6, the cumulative absorbed doses predicted by extrapolation from cycle 1 data were comparable with the observed cumulative absorbed doses at both the group level and the individual patient level. This observation supports the premise that prediction by extrapolation from cycle 1 dosimetry data allows acceptable estimation of cumulative doses over multiple cycles. Similarly, a recently published study showed that it is feasible to simplify the quantitative imaging protocol by performing dosimetry at the first cycle only (38). Predicted cumulative absorbed doses were generally higher than observed cumulative absorbed doses, which is compatible with erring on the side of safety during clinical implementation.

The findings of the present study are not directly comparable with previous dosimetry studies of ^177^Lu-PSMA-617 because of differences in study design, patient selection criteria, and drug manufacturer. Nevertheless, absorbed doses in at-risk organs in the present substudy were consistent with results from previous studies of ^177^Lu-PSMA-617 in patients with mCRPC for the kidneys (10,39), salivary glands (40,41), and red marrow (40,42–44). Data for lacrimal glands have previously been reported with a wide range of values and high variability (10,17,42,43,45). This variability may be due to small volumes of the lacrimal glands and the planar-only analysis. Interference from activity in the nasal mucosa and interpatient variation of actual lacrimal gland mass can further exacerbate this uncertainty. Contouring the lacrimal glands on a CT scan is an extremely challenging task and requires a high-resolution CT image of the orbital area. On the other hand, ranges of normal volumes of lacrimal glands calculated by CT (e.g., for radiotherapy purposes) have been published (26). The findings were also similar but not directly comparable in a prospective dosimetry study of ^177^Lu-PSMA-617 using fewer cycles and lower administered doses in a small number of patients with low-volume metastatic hormone-sensitive prostate cancer (46).

The median duration of ^177^Lu-PSMA-617 exposure in the 30 patients in the present dosimetry substudy was 5.52 mo, similar to the median exposure of 6.9 mo in 529 patients of the pivotal VISION study. The safety profile of ^177^Lu-PSMA-617 was also similar between studies (11). The incidence of bone marrow suppression was similar between the present substudy (36.7%) and the pivotal VISION study (47.4%). In the renal toxicity safety topic of interest, no patients in the dosimetry substudy and 3.4% in the pivotal study experienced TEAEs of CTCAE grade 3 or higher across all treatment cycles. Recently, the results from a prospective registry study showed that ^177^Lu-PSMA-617 did not lead to deterioration in kidney function in patients with mCRPC and kidney impairment (47).

^177^Lu-PSMA-617 is internalized by PSMA-expressing tumor cells and some normal cells that express PSMA. This delivers β-particle radiation with high specificity to prostate cancer cells and PSMA-expressing normal cells and their surrounding microenvironment within an approximate 2-mm range. The duration of exposure depends on the radioactive half-life of ^177^Lu (∼1 wk) and its excretion. The present findings demonstrate that ^177^Lu-PSMA-617 is excreted rapidly into the urine, mainly as the parent compound, with radioactivity in urine at 72 h decreased by approximately 93% from the 0- to 2-h time point. Speculatively, this transient exposure of the kidneys to radioactivity during rapid excretion of unbound ^177^Lu-PSMA-617 may underlie the low incidence of renal toxicity in the present substudy and in the pivotal VISION study (11).

The gold standard for dosimetry comprises a full multiple-time-point curve for every treatment cycle. However, this approach could be logistically, financially, and clinically challenging because of poor health conditions in most patients with mCRPC. Therefore, a simplified dosimetry method was used in cycles 2–6, with imaging at only a single time point (48 h). From a purely dosimetric point of view, this represents a limitation of the study. It can also, however, be regarded as a strength because it minimizes stress for the patient and mitigates the risks of limited clinical capacity by freeing up scanners and staff. The simplified approach has previously been demonstrated to be valid (24). Although it may lead to a slight underestimation of the expected cumulative absorbed doses in the kidneys of patients with mCRPC treated with ^177^Lu-PSMA-617, the simplified approach is recommended as a reliable and appropriate alternative in patients with poor health status (24). An alternative approach to simplify dosimetry would be to use a single-time-point method in all cycles (48). Ultimately, using a single time point in cycle 1 and omitting dosimetry from cycle 2 onward might be the next step toward minimizing complexity, costs, and burden for patients. This will require optimization in patient cohorts larger than that in the present VISION substudy.

The number of patients in this substudy was sufficient for good dosimetric characterization. Given the technical and logistic challenges of dosimetry data acquisition over multiple study sites, the collection of SPECT images that included critical organs at all imaging time points represents a strength of the substudy. Another strength is the multicentric approach: the quantitative imaging calibration procedures were standardized across all 4 participating centers and centralized by the same single operator across all study sites to ensure high-quality data. Other studies have shown that standardization and calibration of quantitative imaging across various sites is feasible and can provide data that are comparable between participating sites (49). The observed renal cumulative absorbed dose was below the historical limit of 23 Gy (12,13). This may present an opportunity to improve the ^177^Lu-PSMA-617 treatment protocol by increasing the dose per cycle or administering extra cycles. Nevertheless, the recommended dose remains 7.4 GBq per cycle for 6 cycles.

## CONCLUSION

This VISION substudy expanded the evidence base for the safety profile of ^177^Lu-PSMA-617, which was shown to extend overall survival and improve radiographic progression-free survival in patients with advanced mCRPC in the pivotal VISION study (11). The use of a simplified single-time-point dosimetry method in cycles 2–6 minimized the burden on patients and scanners and is expected to have little meaningful impact on the reliability of the results. ^177^Lu-PSMA-617 was rapidly eliminated from the body and had a good safety profile, with no patients experiencing renal TEAEs of CTCAE grade 3 or higher. The observed cumulative absorbed doses over 6 cycles of treatment in the kidneys were below the historical EBRT limit of 23 Gy. These data indicate that 44.4 GBq of ^177^Lu-PSMA-617 can safely be administered cumulatively over 6 cycles without inducing renal toxicity. Cumulative absorbed doses were predictable by extrapolation from cycle 1 dosimetry data. Omitting dosimetry from cycle 2 onward may allow adequate assessment of cumulative organ-absorbed doses and thus reduce imaging burden for patients without unacceptably compromising safety. These prospective safety and dosimetry data provide further support for adoption of ^177^Lu-PSMA-617 as a treatment option in clinical practice for patients with mCRPC.

## DISCLOSURE

Ken Herrmann reports consulting or advisory fees from ABX, Aktis Oncology, Amgen, Bayer, BTG, Curium, Endocyte, GE Healthcare, Ipsen, Novartis, Pharma15, Siemens Healthineers, SIRTEX, Sofie Biosciences, Theragnostics, and Y-mAbs. Kambiz Rahbar reports consulting or advisory fees from ABC CRO, ABX, Bayer, Novartis, and SIRTEX. Matthias Eiber reports consulting or advisory fees from Amgen, Bayer, Blue Earth Diagnostics, Lantheus, rhPSMA, and Telix. Richard Sparks and Nicholas Baca are employees of CDE Dosimetry Services, who were contracted by Novartis to perform this analysis. Bernd Krause reports consultant or advisory fees from Bayer, ITM, and Novartis and research funding from Novartis. Michael Lassmann reports research funding from Ipsen and Nordic Nanovector. Jun Tang, Daniela Chicco, Patrick Klein, Lars Blumenstein, and Jean-René Basque are employees of Novartis or own Novartis stocks/shares. This work was supported by Novartis. No other potential conflict of interest relevant to this article was reported.

## Notes

KEY POINTS **QUESTION:** What is the whole-body and organ dosimetry of ^177^Lu-PSMA-617, and can we predict cumulative absorbed radiation doses over multiple cycles of ^177^Lu-PSMA-617 in patients with mCRPC by extrapolation from cycle 1 dosimetry data? **PERTINENT FINDINGS:** ^177^Lu-PSMA-617 has a good overall safety profile and low renal radiotoxicity. Furthermore, data from cycle 1 of patients with mCRPC receiving ^177^Lu-PSMA-617 can be extrapolated to predict cumulative absorbed doses in at-risk organs in additional cycles. **IMPLICATIONS FOR PATIENT CARE:** The omission of dosimetry measurements from cycle 2 onward could reduce patient burden and free scanner and staff capacities, without unacceptably compromising safety.
